# Collectanea Medica: Consisting of Anecdotes, Facts, Extracts, Illustrations, &c.

**Published:** 1821-06

**Authors:** 


### COLLECTANEA MEDICA:

CONSISTING OF

### ANECDOTES, FACTS, EXTRACTS, ILLUSTRATIONS, &c.

*Relating to the History or the Art of Medicine, and the Auxiliary Sciences.*

Floriferis ut apes in saltibus omnia libant,

Omnia nos itidem depascimur aurea dicta.

---

### *Extracts of the Report from the Select Committee of the House of Commons on the Doctrine of the Contagion of the Plague, in* 1819.

*Veneris*, 26° *die Martij*, 1819.

Sir John Jackson, Baronet, in the Chair.

Sir Arthur Brooke Faulkner again called in; and Examined.

I THINK you wish to add to your answer respecting the air of Malta?—I was observing that a high wind, from whatever quarter it blew, was always accompanied with some increase of the number of infected.

Can you give an undoubted proof of the contagion of plague, from any fact that came under your own eye or personal knowledge?—At the military barracks, I think, certainly.

What was it?—The attacks being *consecutive*, as from contact, instead of being *simultaneous*, it was impossible to trace immediate contact with an infected person in barracks, where soldiers were so much together, and lived so gregariously.

If the air was the general cause of plague, must not that have operated as the cause in the instance to which you have alluded?—Certainly not. If the air had operated as the cause, the disease would not have extended consecutively, as I have shown to be the case, but would have been produced through the corps simultaneously. The same would have happened through the population of the island; especially as there were several parts of the island, from local circumstances affecting the air, more exposed to contamination than Valetta.

Is Valetta a lower part of the island than the other parts?—I am not quite sure respecting that; but it is universally allowed to be one of the most healthy parts of the island, the most free from marsh fevers; and, as an instance, I may mention that, in 1801, when marsh fevers were very fatal and numerous, Valetta entirely escaped.

Was there any fever, except on-board the San Nicola, at the time?—I cannot speak with positive certainty, but I think it not unlikely, as vessels from Alexandria, an infected port at that time, were not excluded from entering the harbour contiguous to Valetta.

Whereabout was Salvador Borg's house?—In Strada St. Paulo.

Is that far from Valetta?—It is in Valetta.

Is Valetta the fort?—It is a considerable and well-fortified city, the largest in the island. It is the last spot where one would look for marsh-fevers or diseases of any kind, being perflated with pure sea air in every direction, and the soil being perfectly dry.

Would not the numerous instances you have alluded to, of persons having escaped the plague who had come from the bosom of families afflicted with it, be an inducement, if not a proof, for deeming plague not contagious?—Neither an inducement nor a proof; for we see in other diseases allowed to be contagious, the small-pox for example, that many such escapes have taken place.

Have you any better proof for deeming plague contagious than the preceding observations on my question for deeming it not contagious? —I think that when the whole evidence I have given, respecting the propagation of the plague in a direct line, be well weighed and considered, and when we see that any other supposable cause than contagion is inadequate to account for such extension of the disease in a direct line, the proof is made out as far as presumptive evidence can well render it.

Do you know how many died of the plague in the harbour?—None in the harbour: two, I believe, died on their passage to Malta from Alexandria, and two died in the lazaretto after the infected crew were sent on shore.

You know the ship San Nicola was sent back to Alexandria, without unloading, from Malta?—It was notoriously stated.

And do you know that the goods were landed at Alexandria, and none of the persons took the plague who went with her?—I believe none of the persons who navigated her back took the plague, but arrived in perfect health; farther I know not; I know nothing of her unloading. I have said, there was a report that some bales of goods were missing; but I only speak from public conversation.

Is it not more likely that the persons who went with her to Alexandria should have received the plague, than that a bale of linen carried into Malta from her should have produced the plague?—The ship had undergone a very considerable quarantine; and I heard that means were taken to prevent her from infecting those engaged in her navigation.

Do you suppose her goods were unladen?—I believe not. In every other respect, as far as circumstances would admit, without unlading, I have heard she was expurgated from infection above decks; but I am not certain of this.

Do you believe it would be possible to ventilate the goods without unlading them?—I think it would be impossible.

What number of military were at Malta in 1813?—To the best of my recollection, about 4,000.

What was the greatest number of military afflicted with the plague at the same time?—I am not prepared to answer, as the returns of the whole army were never in my possession; but I can speak as to those under my own care; I have the return of them in my pocket. 4th July, the first case was placed officially under my care; the second was the 8th July.

What is the greatest number at any one time?—I beg to read the return. The third case was the 20th July, the fourth case the 21st

July, the fifth case the 21st July, the sixth case the 23d July, seventh 25th July, the eighth case is the 28th July, the ninth case is the 2d August; I believe that is the last officially under my care.

The sick of all the persons were under your care?—Not of all the forces; some of them were attended by their own regimental surgeons within their own barracks. Those sick only properly belonged to me officially that were sent into a general hospital, although I did attend also in the regimental hospitals. We were at one time so ill off for a general pest-hospital, that medical staff officers were under the necessity of attending plague-cases at the barracks under canvass.

Have you any recollection of the number of soldiers altogether taken ill of the plague?—In all, our army had not hitherto lost above twenty, up to the date before mentioned.

What date?—Up to October.

Were any taken ill after that?—I believe some solitary instances occurred after that. We lost very few in all.

Was any medicine administered which was successful in these cases?—Some articles seemed to be very successful, but there could be no dependence placed upon any.

Would you like to mention any?—Those which I found most beneficial, were the cold affusion and turpentine; to which of the two it is difficult to ascribe the good effects.

Did you use mercury?—I did.

Blood-letting?—Yes, by leeches,—topical blood-letting. Camphor I also used, and several other medicines. Calomel was reported to me as having done some good under the care of the garrison battalion surgeon, but I never found it myself of any use.

Of those infected with the plague, how many do you suppose recovered among the soldiers?—I can only speak as to De Rolle's regiment; I think nearly one half.

Was there any plague at Sasi?—None; nor at some other cassals in the island.

To what do you attribute that?—To infected persons not having made their escape to those cassals.

Was there an order from the commander in chief not to feel the pulses of the plague-patients, under a fine of eighty days' quarantine?—There was. I find now, on referring to my notes, that I have made a mistake: the medical men were interdicted from feeling pulses only under a penalty of quarantine for a *considerable* number of days; the mulct for feeling pulses (even through a tobacco-leaf,) being not less than fifteen or twenty days.

Did you go among or near many persons afflicted with the plague, or did you keep personally from them, except among your own soldiers?—I volunteered my services to examine the patients in the lazaretto, some of whom I saw and examined.

Do you recollect any who were ill in the lazarettos?—I rather think I was prevented from seeing the living cases of plague within their wards, and that I examined more particularly the dead.

Do you consider dead cases capable of producing plague?—From the general opinion which is abroad on this point, I should conceive they are not so liable, if liable at all.

Has the quarantine in Malta been regularly attended to, prior to the breaking-out of the plague?—I have had no opportunities of judging of that; my attention to quarantine-laws was not particularly arrested, till I saw danger in our neighbourhood.

Is it not likely to suppose that cases of plague have frequently arrived in Malta, prior to the breaking-out of the plague?—I understood that it had repeatedly been known in the lazaretto, but was stopped by prompt precaution. I yesterday produced the title-page of a book, in which there was an engraving of a monument to the memory of a grand master in 1743, who had arrested the plague.

Did you remain at Malta after 1813?—I think I left Malta in April or May 1814.

Was there a plague in May 1814, at Malta?—I never heard it recurred in that year.

What was done with the clothes and bedding of the persons who died of the plague?—I understood, generally destroyed; though in some instances not until long after their being infected.

Upon the whole, do you consider the plague to be a disease propagated by contagion, like the small-pox and other eruptive diseases?—I do.

Do you believe it may be so propagated, independent of any influence of the atmosphere?—I believe it may.

Do you consider insulation by the means of quarantine, the most effectual method of preserving against the plague?—By far the most effectual.

Do you know of any cause of communication of plague, besides contagion?—None that will explain its production and dissemination, except a contagion *sui generis.*

Explain your meaning of *sui generis?*—I believe it signifies whatever is quite peculiar to the thing spoken of, consequently the contagion of plague is quite peculiar in producing the plague. Take other examples: the contagion of the small-pox is peculiar in producing small-pox only; the contagion of the measles is peculiar in producing measles only; and so in like manner the contagion of the plague is peculiar to itself, or what is termed specific.

By peculiar, do you only mean that the contagion of the plague will produce plague, and the contagion of the small-pox, small-pox?—I mean that only.

Then you do not allude to the mode of producing the effect?—We know nothing of the *modus operandi* of the contagion; it is quite inscrutible.

Do you wish to state something as to the arrangement for the guards?—I should have mentioned, in my examination on this head, that some part of the police guards were enrolled in the month of July, by the gentlemen placed at the head of the police.

Have you ever heard of the plague in England?—I have.

Mention the year?—I have read of several having visited England, but my recollection respecting them is not distinct: I recollect most of that plague which occurred in the year 1665.

Do you consider Dr. Sydenham's account of the plague as the best? —I think Dr. Sydenham's account did not result from any very patient or philosophical investigation of the disease.

Does Dr. Sydenham consider it the real Levant plague?—I think not; but it is so long since I read the work, that I cannot charge my memory with any of his observations.

Do you consider it yourself as the real plague of the Levant?—I do, decidedly.

Have you ever heard of plague since that period?—I am not prepared to answer that question. Whether it has visited England since or not, I should apprehend, could only be answered by those engaged about the health-office.

If the expurgators of goods at the quarantine establishment have never received plague from opening the bales from the Levant, even those which come with foul bills of health, should you not from thence conclude that there is no matter of infection contained in the bales?—No; the caution enjoined in the operation of expurgation may be sufficiently great to prevent the reception of contagion of plague, even though it existed: the intensity of the contagion may be so blunted by length of time, and by care, as not to be calculated to excite the disease readily.

Has it not astonished you that, for a period of 154 years, there should have been no occurrence of plague in England?—I cannot say it astonishes me more with respect to England than other places. I find it has paid visits, at the distance of long intervals of time, to other countries as well as England; and we do not know enough of what constitutes aptitude in persons to receive the contagion, in order to know under what circumstances the disease must necessarily be produced.

Do you consider the quarantine establishments in England to have been one of the causes of preventing plague from being introduced?—I should think the quarantine establishment, taken in all its bearings, abroad and at home, has had a great share in preventing the introduction of the disease.

Do you suppose the corn is capable of producing infection, and therefore ought to be the subject of the quarantine laws?—On that point I am not able to speak: I do not believe it is considered an article of high susceptibility.

To what articles should you attribute the greatest probability of plague infection?—I cannot be certain, but I should rather think woollen clothes and cotton. Upon this part of the subject I would rather not answer: I am very doubtful with respect to the degrees of susceptibility of different articles.

Were the arrangements made at Malta respecting the plague in 1813, very expensive to government during the plague?—I should think they must have been a very great expence; but I only speak from surmise, concluding from what I saw.

